# Optimizing Cognitive and Physical Gains in Older Adults: Benefits of a Psychomotor Intervention Program Based on Functional Level

**DOI:** 10.3390/medicina61091584

**Published:** 2025-09-02

**Authors:** Hugo Rosado, Jorge Bravo, Armando Raimundo, Joana Carvalho, Catarina Pereira

**Affiliations:** 1 Departamento de Desporto e Saúde, Escola de Saúde e Desenvolvimento Humano, Universidade de Évora, Largo dos Colegiais 2, 7004-516 Évora, Portugal; hrosado@uevora.pt (H.R.); jorgebravo@uevora.pt (J.B.); ammr@uevora.pt (A.R.); 2 Comprehensive Health Research Centre (CHRC), Universidade de Évora, Largo dos Colegiais 2, 7004-516 Évora, Portugal; 3 Faculdade de Desporto, Universidade do Porto, R. Dr. Plácido da Costa 91, 4200-450 Porto, Portugal; mjoanacarvalho@reit.up.pt; 4 CIAFEL-Research Centre in Physical Activity, Health and Leisure, Universidade do Porto, R. Dr. Plácido da Costa 91, 4200-450 Porto, Portugal

**Keywords:** aging, balance, community-dwelling, lower-body strength, processing speed

## Abstract

*Background and Objectives:* Aging is associated with heterogeneous declines in cognitive and physical functions, yet little is known about how baseline functional levels influence the effectiveness of intervention programs. This study analyzed the effects of a psychomotor intervention program on cognitive and physical functions in community-dwelling older adults, considering their baseline functional levels. *Materials and Methods:* Fifty-one participants (75.4 ± 5.6 years) were divided into an experimental group, which underwent the intervention, and the control group. The experimental group was further divided into lower-functioning (LFG) and higher-functioning (HFG) subgroups based on baseline assessments. Participants were assessed at baseline, 24-week post-intervention, and after a 12-week follow-up. *Results:* Significant improvements were observed in both experimental subgroups, particularly LFG, in processing speed, executive functions, reaction time, attention, lower-body strength, balance, and mobility (*p* < 0.05). Cognitive gains persisted post-follow-up, while physical gains were reversed, especially in LFG (*p* < 0.05). Effect sizes ranged from medium to large in both lower- and higher-functioning groups. *Discussion:* The intervention improved cognitive and physical functions in both lower- and higher-functioning groups. Although older and less educated, the lower-functioning group showed greater gains but also more decline after follow-up. These findings emphasize that older adults with diverse baseline functional levels can improve substantially, highlighting the need for tailored psychomotor interventions to maximize benefits and address individual variability. The study was registered at ClinicalTrials.gov (NCT03446352).

## 1. Introduction

Demographic projections indicate a significant increase in life expectancy between 2021 and 2100 [1]. While encouraging, this trend also implies greater risk of age-related declines in functional capacity, often leading to loss of independence [2]. Specifically, changes in cognitive functions—particularly processing speed, executive functions, reaction time, and attention—can impair daily activities [3,4,5]. Similarly, reductions in lower-body strength, balance, and mobility compromise physical functionality and increase fall risk [6,7].

Nevertheless, cognitive and physical impairments can be attenuated through targeted training. Recent meta-analyses show that interactive cognitive–motor (ICM) training, which combines simultaneous cognitive and motor tasks (e.g., dual-task paradigms), yields meaningful improvements in both domains [3,7]. The synergy between cognition and physical activity helps explain these benefits. However, evidence also shows that such gains, particularly in physical function, may diminish once training ceases (detraining) [8], whereas cognitive benefits may persist longer [9]. Understanding these post-intervention changes is essential for designing sustainable programs for older adults.

Effective interventions to promote or preserve functionality in older adults are therefore crucial. Psychomotor interventions, which use movement and body as mediators, have shown promise in enhancing neurocognitive and sensorimotor functions [10,11]. Incorporating ICM principles, they may surpass single-domain approaches such as exercise-only or computerized cognitive training [12].

However, substantial inter-individual variability exists in both cognitive and physical capacities among community-dwelling older adults. Cognitive reserve, shaped by personal, social, and environmental factors, explain some variability in cognitive performance [13]. Likewise, sedentary behavior or activity level affects physical function and responsiveness to training [2]. Prior research indicates that baseline performance can moderate training outcomes, with two main patterns emerging: a compensation effect, in which individuals with lower baseline performance improve more due to greater room for enhancement, and a magnification effect, in which individuals with higher baseline performance benefit most by leveraging more efficient cognitive and motor resources [14]. Evidence for each pattern is mixed, with some studies reporting greater gains among higher-functioning individuals, while others show the opposite trend [14,15,16].

Identifying which individuals benefit most from training is therefore essential. Few studies have classified community-dwelling older adults by baseline functional level [2,17], and those that did often presented methodological limitations in adequately addressing cognitive and physical variability (e.g., cross-sectional design). Moreover, research on cessation training remains scarce, with limited evidence on how gains are maintained or lost after program termination [8,9]. To our knowledge, apart from the study protocol by Mack and colleagues [18], which investigated individual differences only during the 12-week training phase, no ICM intervention has examined whether baseline functional level influences both the magnitude and retention of cognitive and physical gains. This gap limits the development of tailored intervention strategies that could maximize benefits across heterogeneous older adult populations.

Thus, the present study aimed to analyze the training and training cessation effects of a psychomotor intervention program on cognitive and physical functions in community-dwelling older adults considering their functional level at baseline.

## 2. Materials and Methods

### 2.1. Study Design and Participants

This 24-week single-blinded clinical trial was conducted between March 2018 and January 2019. In this design, the assessor responsible for all evaluations was blinded to group allocation. Participants were allocated to one of two groups: an experimental group (EG), which underwent a psychomotor intervention program, or a control group (CG), which continued their usual daily activities.

The study adhered to the Standard Protocol Items: Recommendations for Interventional Trials (SPIRIT) guidelines “https://www.spirit-statement.org/publications-downloads/ (accessed on 19 May 2025)” and included an intervention description following the TIDieR checklist “https://www.equator-network.org/reporting-guidelines/tidier/ (accessed on 19 May 2025)”. This study forms part of the registered randomized controlled trial (ClinicalTrials.gov: NCT03446352; registration date: 26 February 2018). Related analyses from this trial have been published elsewhere [19,20]; however, the present study provides complementary analyses addressing a novel research question.

Community-dwelling older adults from Portugal were recruited via pamphlets distributed at senior universities and recreational centers and through verbal invitations. Inclusion criteria were: (a) age ≥ 65 years; and (b) moderate or higher physical independence (≥18 points on the Composite Physical Function scale) [21]. Exclusion criteria were: (a) cognitive impairment; (b) dependent mobility.

**Figure 1:**
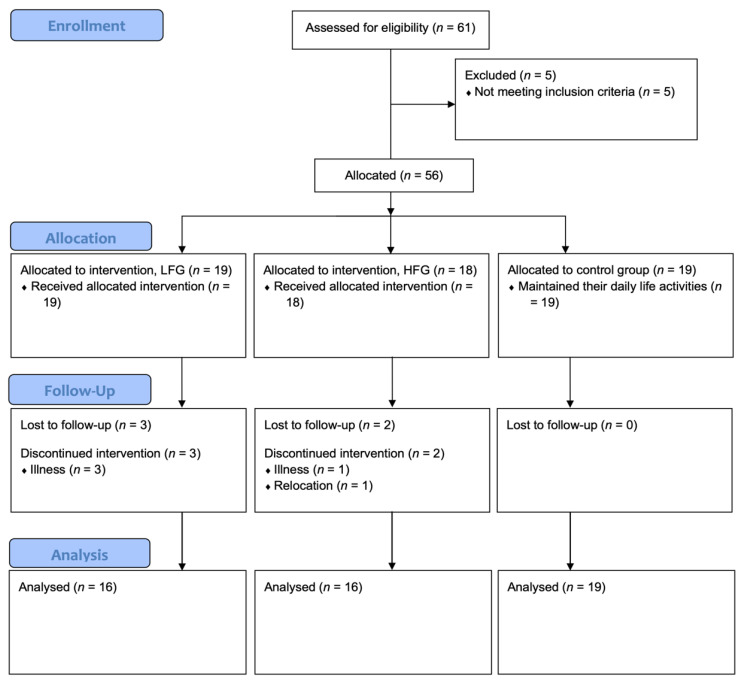
Flow diagram.

Of 61 eligible participants, five were excluded for not meeting inclusion criteria (Figure 1). The remaining 56 participants were allocated to either the EG or CG, with those in the EG further subdivided into a lower-functioning group (LFG) and a higher-functioning group (HFG), based on their baseline values. During the intervention, five participants in the LFG and HFG dropped out due to illness (i.e., musculoskeletal and respiratory conditions) or relocation, leaving 51 completers (LFG: *n* = 16; HFG: *n* = 16; CG: *n* = 19). Of these, 42 were women and nine were men.

Ethical approval was granted by the institutional research ethics committee on human health and well-being (reference no. 16012; approval date: 19 May 2016), and the study complied with the Declaration of Helsinki. Written informed consent was obtained from all participants.

### 2.2. Procedures

Assessments were conducted at baseline (m_1_), after 24 weeks of intervention (m_2_), and after a 12-week no-intervention follow-up (m_3_). Cognitive and questionnaire assessments were conducted in a quiet room, while physical assessments took place in a laboratory. All participants were evaluated individually by the same trained psychomotor rehabilitation professional, who remained blinded to group allocation throughout the study. During the follow-up, participants were instructed to maintain their usual activities and not integrate any exercise program.

### 2.3. Outcome Measures

Processing speed. Assessed with the Trail Making Test-A (TMT-A). Participants were asked to draw lines on paper connecting 25 numbers by order [22]. Time (s) to completion was recorded.

Executive functions. Assessed with the Trail Making Test-B (TMT-B). The task was similar to the TMT-A task, in which participants were required to alternately connect numbers and letters in ascending order (i.e., 1-A-2-B, etc.) [22]. Time (s) to completion was recorded.

Reaction time. Assessed using the Deary–Liewald reaction time task [23], which included Simple (SRT) and Choice (CRT) tasks. Median reaction times (ms) were recorded.

Selective and sustained attention. Assessed with the d2 Test of Attention [24]. For each of the 14 lines of the test, participants had 20 s to identify and mark the letter “d” with two dashes as quickly as possible. Performance measurements included the items recognized correctly (n) and the concentration index (n).

Lower-body strength. Measured with the 30-s chair stand test [21]. Number of correct stands completed in 30 s was recorded.

Multidimensional balance. Measured with the Fullerton Advanced Balance (FAB) scale [25]. This 10-item scale ranged from 0 points (worst) to 40 points (best).

Mobility. Measured with the Timed Up and Go (TUG) test [21]. The best time (s) of two trials was recorded.

#### Complementary Outcomes Measures

Exercise intensity was monitored using the Borg Rating of Perceived Exertion (RPE) scale, which ranged from 6 (very, very light) to 20 points (very, very hard) [26]. Participant satisfaction was assessed using the Caregiver Treatment Satisfaction (CTS) questionnaire with scores ranging from 1 (extremely dissatisfied) to 5 points (extremely satisfied) [27]. Sociodemographic data were collected via interview based on a script. Body mass (kg) (electronic scale: Seca 760, Hamburg, Germany) and height (m) (stadiometer: Seca 206, Hamburg, Germany) were collected to calculate the body mass index (kg/m^2^). Physical independence was assessed with the 12-item CPF scale, classifying participants as low (<18), moderate (18–23), or high (24) functioning [21]. Habitual physical activity was measured with the short-form International Physical Activity Questionnaire (IPAQ), calculating total MET-min/week [28].

### 2.4. Interactive Cognitive-Motor Program

The psychomotor intervention (75 min/session, 3×/week on alternate days) was delivered by the same psychomotor specialist (master’s degree in psychomotricity). Sessions (max. 10 participants) were rescheduled if a participant missed three consecutive sessions, ensuring ≥80% attendance. All sessions took place in the gerontopsychomotricity laboratory.

The program emphasized ICM stimulation (e.g., dual-task activities), with progressive increases in complexity and intensity (~13 on the RPE scale) in line with American College of Sports Medicine guidelines [29].

#### Psychomotor Intervention Program

This program used movement and corporality (e.g., body awareness) as the main principles. Each session comprised five phases: (a) initial dialogue (~5 min), (b) global activation (~10 min), (c) main phase (~50 min), (d) cool-down (~5 min), and (e) final dialogue (~5 min The main phase followed joint rotations and neurophysiological activation and featured neurocognitive, motor, and sensory activities promoting simultaneous cognitive, perceptual, and motor stimulation (e.g., moving around cones with a fitball while naming animals/countries; balance and postural changes activities with regressive countdowns; naming body parts paired with movements). Cool-down involved relaxation with massage balls. Participants recorded perceived intensity and satisfaction after each session.

### 2.5. Statistical Analysis

Analyses were conducted using SPSS v26.0 and JASP v2.2.5. Significance was set at *p* < 0.05.

For the EG, participants were classified as LFG or HFG for each outcome based on the <50th or ≥50th percentile at baseline. Thus, the lower- and higher-functioning experimental groups differed according to each variable, although showing similar characteristics. The CG was not stratified because their variation showed no meaningful differences between potential LFG and HFG subgroups.

Delta (∆) and %Delta (%∆) were calculated as: ∆ = moment_x_ − moment_x−1_, and %∆ = [(moment_x_ − moment_x−1_)/moment_x−1_] × 100). Descriptive data are reported as mean ± standard deviation.

Assumptions of normality and homogeneity of variance were not met; thus, nonparametric tests were used. Between-group comparisons were performed using the Mann-Whitney U and the Kruskal-Wallis tests, followed by pairwise post hoc tests. For within-group changes, the Wilcoxon signed-rank test was used to test whether the median difference (delta) was significantly different from zero. In addition, within-group comparisons were performed using the Friedman test for both experimental LF or HF groups.

Finally, the effect size (ES) for the magnitude of the treatment effect was calculated for the within-group and between-group comparisons following the instructions for nonparametric tests [30]. The ES was computed as r = (Z/√N), and interpreted as small (0.10), medium (0.30), or large (0.50) [31].

## 3. Results

### 3.1. Sociodemographic Characteristics and Response Rates

No significant differences were found between the experimental and control groups in baseline sociodemographic characteristics, physical independence, or habitual physical activity, *p* > 0.05. Participants ranged from 66 to 89 years, with women predominating in both groups. Table 1 presents the baseline sociodemographic characteristics of the lower- and higher-functioning groups.

Overall, 51 participants completed the study, corresponding to a dropout rate of 9.8%. The five participants who withdrew had similar baseline characteristics to those who completed the study, and none of the withdrawals were attributable to adverse effects of the intervention. Attendance at the psychomotor intervention sessions was 83.3% (out of 75 sessions). Reported effort levels (RPE: 13.0 ± 0.3) aligned with the planned intensity, and satisfaction was rated as “extremely satisfied” (CTS: 5.0 ± 0.0)

### 3.2. Descriptive Results

Table 2 presents the descriptive results for all variables across the lower- and higher-functioning experimental groups and the CG. At baseline, no significant differences were observed between the experimental and control groups.

**Figure 2:**
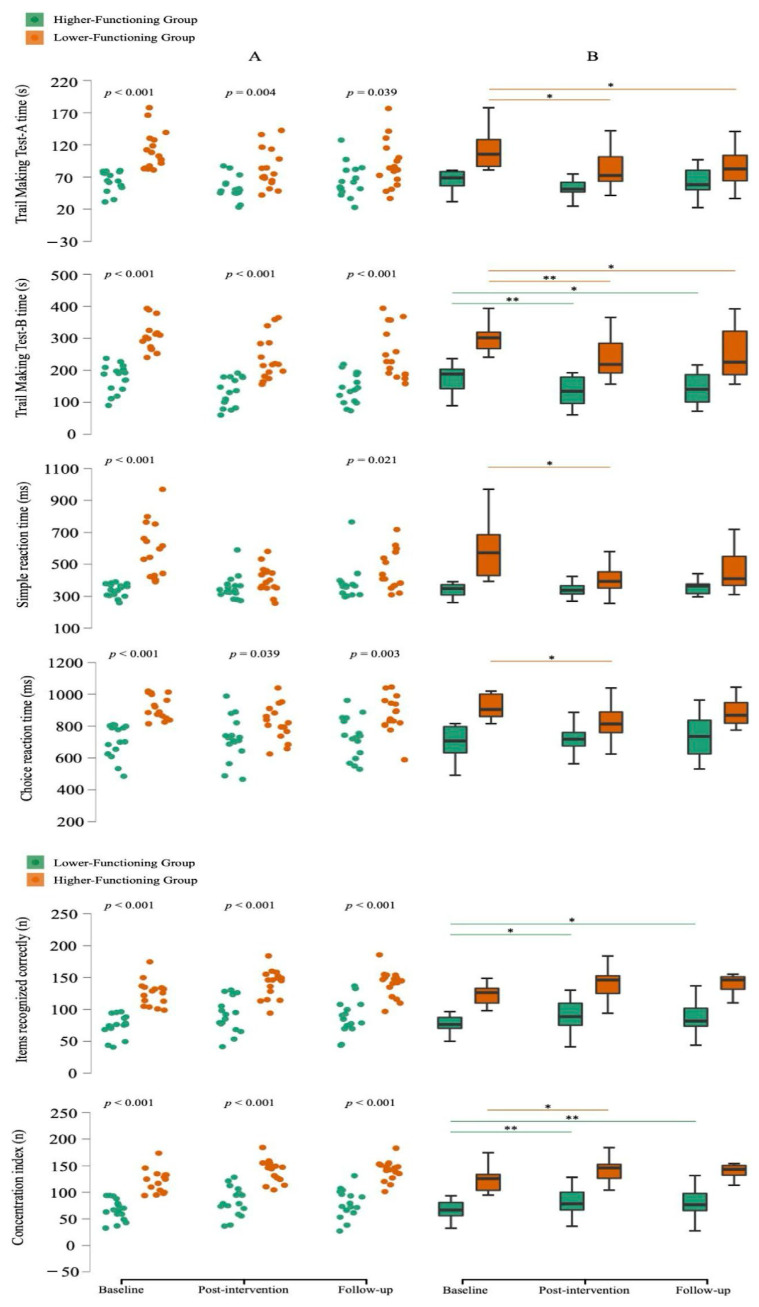
(**A**): Jitter plots comparisons between the baseline, post-intervention, and follow-up evaluations; *p*-value: significant differences between groups. (**B**): Box plots (median, interquartile range, minimum, and maximum) comparisons between the baseline, post-intervention, and follow-up evaluations; Median values are represented by bold horizontal lines, and the first and third quartiles are indicated by the lower and upper hinges, respectively. * significant differences within groups, *p* < 0.05; ** significant differences within groups, *p* < 0.001. Lower-functioning group (*n* = 16); Higher-functioning group (*n* = 16).

Notably, in the CG, changes from baseline to post-intervention and from post-intervention to follow-up were negligible for most variables (Δ ≈ 0). Exceptions included ‘Simple reaction time’, ‘Choice reaction time’, and ‘Mobility’, which showed significant declines in performance from baseline to post-intervention; however, from post-intervention to follow-up, the CG improved in ‘Choice reaction time’ (*p* < 0.05). Consequently, subsequent analyses focused on the experimental lower- and higher-functioning groups (Figure 1 and Figure 2).

#### 3.2.1. Cognitive Function

For cognitive function (Figure 2), both LFG and HFG showed significant within-group improvements from baseline to post-intervention in processing speed, executive functions, reaction time, and attention. Improvements in “TMT-A time”, “Simple reaction time”, “Choice reaction time”, and “Concentration index” were more pronounced in LFG, while HFG improved more in “TMT-B time” and “Items recognized correctly”. Effect sizes (ES) ranged from medium to large in LFG (0.40–0.62) and large in HFG (0.58–0.61).

Improvements in “TMT-A time”, “TMT-B time”, “Items recognized correctly”, and “Concentration index” were maintained at follow-up (m_3_ vs. m_2_), with medium ES in LFG (0.43–0.48) and large ES in HFG (0.58–0.62).

Between-group comparisons at post-intervention and follow-up revealed significant differences in all cognitive variables except “Simple reaction time” at post-intervention, with ES ranging from medium to large (0.37–0.81).

#### 3.2.2. Physical Function

**Figure 3:**
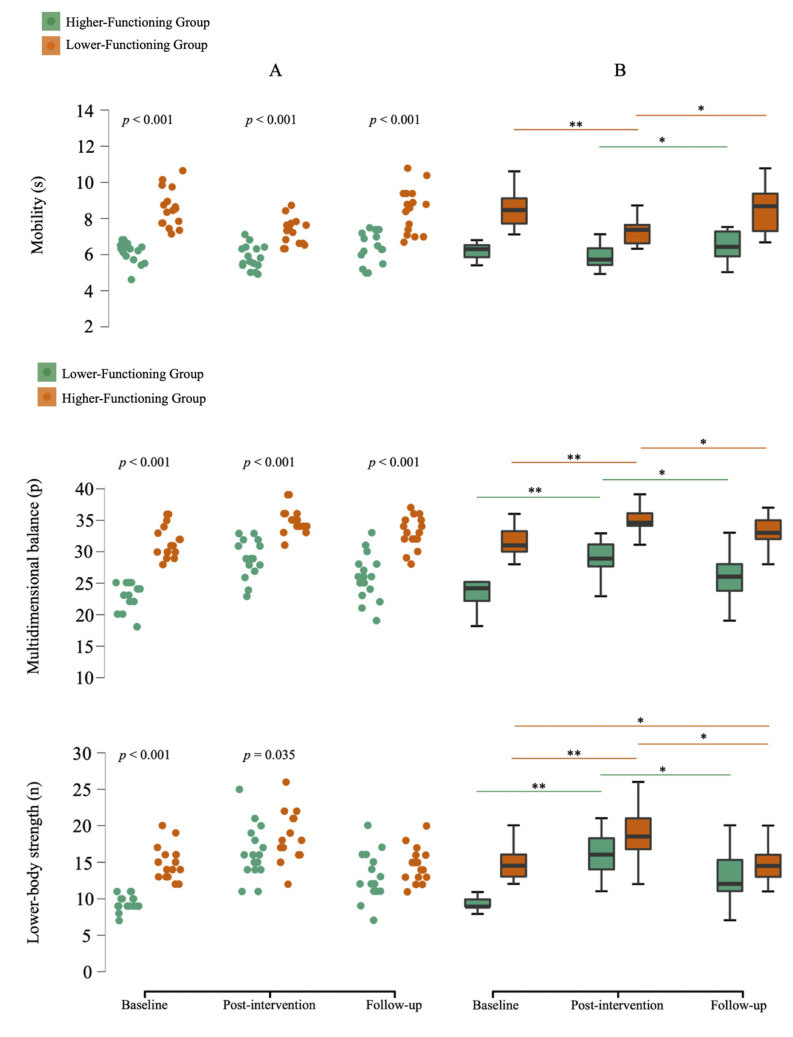
(**A**): Jitter plots comparisons between the baseline, post-intervention, and follow-up evaluations; *p*-value: significant differences between groups. (**B**): Box plots (median, interquartile range, minimum, and maximum) comparisons between the baseline, post-intervention, and follow-up evaluations; Median values are represented by bold horizontal lines, and the first and third quartiles are indicated by the lower and upper hinges, respectively. * significant differences within groups, *p* < 0.05; ** significant differences within groups, *p* < 0.001. Lower-functioning group (*n* = 16); Higher-functioning group (*n* = 16).

For physical function (Figure 3), both LFG and HFG improved from baseline to post-intervention in “Lower-body strength” and “Multidimensional balance”, while improvements in “Mobility” were observed only in LFG. Effect sizes were large in both groups (LFG: 0.57–0.62; HFG: 0.60–0.61). At follow-up, most physical gains had diminished in both groups; however, LFG retained a significant improvement in “Lower-body strength” compared to baseline (37.1%; ES = 0.53). The respective ES at follow-up remained large in LFG (0.60–0.62) and medium-to-large in HFG (0.48–0.60).

Between-group comparisons at post-intervention and follow-up showed significant differences in all physical variables except Lower-body strength at follow-up, with medium-to-large ES at post-intervention (0.37–0.81) and large ES at follow-up (0.69–0.76).

## 4. Discussion

This study showed that a 24-week psychomotor intervention improved cognitive and physical functions in community-dwelling older adults, both with lower (LFG) and higher (HFG) functional levels at baseline. The most significant gains were observed in the LFG, composed predominantly of older adults with lower educational level. These findings challenge the assumption that individuals with lower baseline functioning benefit less from training [15,16], and instead align more closely with the compensation hypothesis, as lower-functioning participants exhibited the largest improvements despite potentially having fewer cognitive and physical resources at baseline [14]. Conversely, HFG participants maintained higher absolute performance across most outcomes, reflecting aspects of the magnification hypothesis, whereby those with higher baseline levels can consolidate gains more effectively [14]. Overall, these results underscore the importance of considering baseline functional level when designing interventions, suggesting that tailored ICM programs can help counteract initial disadvantages and optimize benefits for populations at greater risk of functional decline.

After a 12-week no-intervention follow-up, the LFG maintained improvements in processing speed and executive functions, while the HFG retained gains in executive functions and attention. However, physical gains in strength, balance, and mobility regressed in both groups, especially in the LFG. The LFG consistently underperformed compared to the HFG across all time evaluations. Treatment effects remained medium to large at follow-up. Post-intervention and follow-up results showed the HFG outperformed the LFG in most outcomes, except for SRT time and lower-body strength, with between-group effects ranging from medium to large.

In contrast, the CG maintained or declined in performance, highlighting the importance of interventions for older adults. These findings can guide professionals in tailoring interventions based on initial functional levels.

Before delving deeper into our results, other relevant factors deserve attention. Exercise intensity may not be crucial for cognitive and physical benefits, as shown in Tse, Wong, and Lee’s systematic review [32], who found low to moderate intensity sufficient in healthy older adults. This aligns with our results, as both groups engaged in similar intensity activities. Nevertheless, the program benefitted both functional levels, supporting World Health Organization’s call for proactive aging strategies [33]. The program’s design—challenging, enjoyable, and social—also aligns with factors that enhance effectiveness [3,34].

Cognitive gains were notable, especially in the LFG, with significant improvements in processing speed, executive functions, reaction time, and attention. Given the limited studies controlling for baseline function, comparisons must be cautious. However, gains in processing speed (LFG) and executive functions (both groups) surpassed those in previous ICM studies [9,35,36]. Similar to Olyaei et al.’s study [9], our LFG maintained improvements in processing speed and executive functions. Our follow-up results also echo Eggenberger et al. [37], showing long-term executive function retention. Sustained cognitive gains may be linked to longer interventions (≥24 weeks), transfer effects, and neurophysiological adaptations.

The LFG also improved in SRT, contrasting with Vaughan et al. [4], who found no changes in SRT or CRT, likely due to their motor-focused training. Both groups improved in attention, especially the HFG, paralleling Linde & Alfermann study [5], though our HFG retained those gains at follow-up, unlike in their study.

Cognitive reserve may partly explain these patterns, as functional level influences improvement potential [38]. While lower-functioning individuals may benefit from a higher learning curve, higher-functioning ones may leverage neuroplasticity more efficiently [38]. Therefore, neuroplasticity also plays a crucial role, as it facilitates cognitive function enhancements and serves as a protective mechanism against brain changes [13,39,40], supporting the “use it or lose it” principle [39].

In terms of physical function, both groups improved in strength and balance, and the LFG also in mobility. Yet, unlike cognitive improvements, these gains were not maintained post-intervention, especially in the LFG. Despite moderate to high baseline physical independence (CPF scale), individual variability remained, suggesting our functional-level classification may better capture change potential.

LFG results for strength and mobility align with Blasco-Lafarga et al. [8]. Both groups improved in 30-CST post-intervention but regressed more at follow-up. Although the LFG had smaller TUG gains post-intervention, they retained better mobility than at baseline. HFG mobility changes align with Brahms et al. [40], reinforcing the need for longer and more intense training (e.g., strength/power exercises), which were not part of our program.

As for balance, our use of the FAB scale mirrors Cho et al. Cho et al. [6], where both high- and low-fall-risk groups improved. Our LFG improved 25.8% in the FAB, suggesting greater benefit for those with poorer baseline balance. Still, their balance-focused eight-week program limits comparability. Our results also support Blasco-Lafarga et al. [8], suggesting that physical gains are more vulnerable to regression post-intervention.

In summary, our results reinforce Pauwels et al. [41], showing that cognitive and motor learning can occur at any age. The study is novel in stratifying participants by baseline functional level, examining both cognitive and physical outcomes, and evaluating training cessation effects, addressing a gap identified in prior research. The findings support lifelong neuroplasticity and the potential of older adults to maintain independence and quality of life [41]. Importantly, they highlight that older adults with lower baseline functional levels can achieve substantial gains, while higher-functioning participants may require more complex or intensified stimuli to optimize outcomes. These results underscore the practical value of tailoring ICM programs to participants’ initial functional capacities, maximizing training benefits, preventing functional decline, and promoting independence and quality of life in community-dwelling older adults. This supports policy-making for aging populations and individualized program design. Future research should explore memory-related gains or replicate our design with institutionalized older adults.

This study has strengths and limitations. To our knowledge, it is the first to include a CG and follow-up while accounting for baseline functional levels. Limitations include the small sample size and unequal EG and CG sizes. As in other studies [6], female participants predominated. Lastly, our classification into LFG and HFG was based on sample median values, not population-based cutoffs [42], which may limit generalizability for outcomes such as mobility. Further research is needed to confirm these findings.

## 5. Conclusions

This study showed that a psychomotor intervention significantly improved cognitive and physical functions in both lower- and higher-functioning older adults. Notably, the LFG—despite being older and having lower educational attainment—demonstrated greater improvements, particularly in processing speed, executive functions, lower-body strength, balance, and mobility. However, they also experienced greater physical declines after training cessation, indicating both high responsiveness and vulnerability to detraining. Cognitive gains were maintained in both groups, while physical improvements regressed, particularly in the LFG. Still, the HFG retained overall higher performance levels. These findings reinforce the importance of tailoring psychomotor interventions to individuals’ baseline functional levels. They also highlight the potential for meaningful gains among older adults with lower functionality and education, who are often overlooked in traditional training approaches. Personalized ICM programs can better address this diversity, promoting more effective and sustained improvements in cognitive and physical functions among community-dwelling older adults. Finally, these findings extend prior investigations by showing that baseline functional level not only shapes training responsiveness but also influences the durability of benefits.

## Figures and Tables

**Table 1:** Sociodemographic characteristics of LFG and HFG at baseline.

	Experimental LFG Mean ± SD	Experimental HFG Mean ± SD	*p*-Value
Cognitive function			
Trail Making Test-A (time) (s)			
Age (years)	77.9 ± 4.3	71.1 ± 3.9	<0.001
Educational level (years)	4.9 ± 2.1	7.3 ± 3.3	0.051
Trail Making Test-B time (s)			
Age (years)	75.1 ± 5.1	73.9 ± 5.6	0.491
Educational level (years)	4.8 ± 1.8	7.3 ± 3.5	0.035
Simple reaction time (ms)			
Age (years)	74.6 ± 6.0	74.4 ± 4.7	0.985
Educational level (years)	5.1 ± 2.3	7.0 ± 3.4	0.160
Choice reaction time (ms)			
Age (years)	75.6 ± 5.3	73.4 ± 5.3	0.270
Educational level (years)	4.5 ± 1.7	7.6 ± 3.2	0.010
Items recognized correctly (n)			
Age (years)	76.1 ± 5.3	72.9 ± 5.0	0.110
Educational level (years)	5.1 ± 2.4	7.0 ± 3.3	0.119
Concentration index (n)			
Age (years)	75.8 ± 4.9	73.2 ± 5.6	0.160
Educational level (years)	4.4 ± 1.5	7.8 ± 3.2	0.002
Physical function			
Lower-body strength (n)			
Age (years)	73.5 ± 5.6	75.5 ± 5.0	0.323
Educational level (years)	6.4 ± 3.4	5.7 ± 2.5	0.590
Multidimensional balance (points)			
Age (years)	75.9 ± 6.1	73.1 ± 4.1	0.138
Educational level (years)	5.2 ± 3.3	6.9 ± 2.4	0.026
Mobility (s)			
Age (years)	75.4 ± 5.7	73.6 ± 4.9	0.323
Educational level (years)	5.1 ± 3.1	7.1 ± 2.6	0.026

Abbreviations: LFG, low-functioning group (*n* = 16); HFG, high-functioning group (*n* = 16); SD, standard deviation; Significant differences between groups, *p* < 0.05.

**Table 2:** Descriptive results of the participant’s cognitive and physical functions.

	Baseline (Mean ± SD)	∆ Post-Intervention (Mean ± SD)	∆ Follow-Up (Mean ± SD)
Cognitive function			
Trail Making Test-A (time) (s)			
Experimental LFG	111.9 ± 29.9 ^a^	−29.3 ± 21.6 ^#^	6.6 ± 22.5
Experimental HFG	64.6 ± 16.0	−10.2 ± 13.9 ^#^	9.6 ± 17.8 ^#^
CG	84.4 ± 39.7 ^b^	−3.9 ± 25.6 ^b^	−2.9 ± 16.7
Trail Making Test-B time (s)			
Experimental LFG	304.4 ± 47.4 ^a^	−66.6 ± 42.1 ^#,a^	11.7 ± 38.0
Experimental HFG	174.4 ± 43.1	−43.6 ± 38.4 ^#^	10.5 ± 26.6
CG	207.2 ± 80.4 ^b^	−3.3 ± 44.8 ^b,c^	−13.8 ± 31.4 ^b^
Simple reaction time (ms)			
Experimental LFG	588.3 ± 171.5 ^a^	−183.3 ± 172.4 ^#,a^	51.4 ± 105.8
Experimental HFG	339.9 ± 40.3	17.5 ± 73.8	21.5 ± 49.5
CG	418.7 ± 143.6 ^b^	41.8 ± 81.5 ^#,b^	3.1 ± 82.6
Choice reaction time (ms)			
Experimental LFG	1061.5 ± 89.2 ^a^	−117.4 ± 116.8 ^#,a^	61.8 ± 111.2 ^#^
Experimental HFG	800.8 ± 125.0	20.6 ± 95.6	7.1 ± 91.3
CG	916.4 ± 172.7 ^b^	99.5 ± 158.6 ^#,b^	−53.1 ± 112.0 ^#,b^
Items recognized correctly (n)			
Experimental LFG	73.8 ± 17.3 ^a^	16.4 ± 24.2 ^#^	−3.2 ± 11.0
Experimental HFG	124.5 ± 19.9	15.8 ± 11.9 ^#^	0.4 ± 6.7
CG	95.5 ± 34.8 ^c^	0.5 ± 16.2 ^c^	3.9 ± 10.3
Concentration index (n)			
Experimental LFG	65.9 ± 19.8 ^a^	16.1 ± 26.5 ^#^	−3.1 ± 11.7
Experimental HFG	121.8 ± 21.5	18.6 ± 12.6 ^#^	−0.5 ± 10.8
CG	89.8 ± 38.1 ^c^	−0.6 ± 21.3 ^c^	6.8 ± 14.3
Physical function			
Lower-body strength (n)			
Experimental LFG	9.4 ± 1.1 ^a^	6.9 ± 3.5 ^#^	−3.4 ± 1.7 ^#^
Experimental HFG	14.9 ± 2.3	3.8 ± 2.6 ^#^	−4.1 ± 2.4 ^#^
CG	12.7 ± 3.2 ^b^	−0.6 ± 2.1 ^b,c^	−0.4 ± 2.2 ^b,c^
Multidimensional balance (points)			
Experimental LFG	23.1 ± 2.1 ^a^	5.9 ± 2.1 ^#^	−3.1 ± 1.7 ^#^
Experimental HFG	31.6 ± 2.5	3.3 ± 1.6 ^#^	−1.9 ± 1.5 ^#^
CG	29.7 ± 3.2 ^b^	−0.2 ± 1.8 ^b,c^	−0.6 ± 1.4 ^b^
Mobility (s)			
Experimental LFG	8.5 ± 1.1 ^a^	−1.3 ± 1.0 ^#,a^	1.2 ± 1.0 ^#^
Experimental HFG	6.1 ± 0.6	−0.3 ± 0.6 ^#^	0.6 ± 0.7 ^#^
CG	7.0 ± 1.5 ^b^	0.5 ± 0.9 ^#,b^	0.4 ± 1.0 ^b^

Abbreviations: SD, standard deviation; Experimental LFG: experimental lower-functioning group (*n* = 16); Experimental HFG: experimental higher-functioning group (*n* = 16); CG: control group (*n* = 19); ^#^ ∆ different from 0, *p* < 0.05; ^a^ significant differences between Experimental LFG and Experimental HFG, *p* < 0.05; ^b^ significant differences between CG and Experimental LFG, *p* < 0.05; ^c^ significant differences between CG and Experimental HFG, *p* < 0.05.

## Data Availability

The datasets used and/or analyzed during the current study are available from the corresponding author upon reasonable request.
